# Rubbery-Modified CFRPs with Improved Mode I Fracture Toughness: Effect of Nanofibrous Mat Grammage and Positioning on Tanδ Behaviour

**DOI:** 10.3390/polym13121918

**Published:** 2021-06-09

**Authors:** Emanuele Maccaferri, Laura Mazzocchetti, Tiziana Benelli, Tommaso Maria Brugo, Andrea Zucchelli, Loris Giorgini

**Affiliations:** 1 Department of Industrial Chemistry “Toso Montanari”, University of Bologna, Viale Risorgimento 4, 40136 Bologna, Italy; emanuele.maccaferri3@unibo.it (E.M.); tiziana.benelli@unibo.it (T.B.); loris.giorgini@unibo.it (L.G.); 2 Interdepartmental Center for Industrial Research on Advanced Applications in Mechanical Engineering and Materials Technology, CIRI-MAM, University of Bologna, Viale Risorgimento 2, 40136 Bologna, Italy; tommasomaria.brugo@unibo.it; 3 Department of Industrial Engineering, University of Bologna, Viale Risorgimento 2, 40136 Bologna, Italy

**Keywords:** damping, delamination, interlaminar fracture toughness, matrix toughening, rubber, nanofiber, epoxy carbon fiber composite, dynamic mechanical analysis (DMA), mechanical properties, tensile testing

## Abstract

Carbon Fiber Reinforced Polymers (CFRPs) are widely used where high mechanical performance and lightweight are required. However, they suffer from delamination and low damping, severely affecting laminate reliability during the service life of components. CFRP laminates modified by rubbery nanofibers interleaving is a recently introduced way to increase material damping and to improve delamination resistance. In this work, nitrile butadiene rubber/poly(ε-caprolactone) (NBR/PCL) blend rubbery nanofibrous mats with 60 wt% NBR were produced in three different mat grammages (5, 10 and 20 g/m^2^) via single-needle electrospinning and integrated into epoxy CFRP laminates. The investigation demonstrated that both mat grammage and positioning affect CFRP tanδ behaviour, evaluated by dynamic mechanical analysis (DMA) tests, as well as the number of nano-modified interleaves. Double cantilever beam (DCB) tests were carried out to assess the mat grammage effect on the interlaminar fracture toughness. Results show an outstanding improvement of G_I,*R*_ for all the tested reinforced laminates regardless of the mat grammage (from +140% to +238%), while the effect on G_I,*C*_ is more dependent on it (up to +140%). The obtained results disclose the great capability of NBR/PCL rubbery nanofibrous mats at improving CFRP damping and interlaminar fracture toughness. Moreover, CFRP damping can be tailored by choosing the number and positioning of the nano-modified interleaves, besides choosing the mat grammage.

## 1. Introduction

Composite laminates, such as high-performance Carbon Fiber Reinforced Polymers (CFRPs), may need improved properties to replace conventional metallic materials in specific fields. In particular, the main weakness is due to their laminar structure, representing itself an intrinsic vulnerability. Indeed, laminates commonly fail by delamination, promoted either by impacts or fatigue. Such a detrimental effect can be mitigated by increasing material damping, thus allowing the extension of the component service life by reducing the propagation of mechanical solicitations and unwanted vibrations.

Matrix toughening has for a long time been the approach to increase interlaminar fracture toughness. The simplest methods involve mixing uncrosslinked “liquid” rubber with resin precursors or the addition of already crosslinked rubbery particles, adding up to 20 wt% of toughener [1,2,3,4,5]. This usually implies the modification of the whole resin bulk, requiring a new formulation for each specific case. Another viable solution is the integration of viscoelastic films between laminae [6,7,8]. While it represents a simple and economical solution, the use of bulk layers negatively affects laminate stiffness and strength, besides determining significant weight and size increase [9].

For these reasons, a targeted and scarcely invasive modification is welcome to decrease the impact on the overall composite properties. In recent years, nano-modifiers are gaining growing interest thanks to their outstanding properties, high versatility and the need for low quantities to achieve the desired effects [10,11,12]. Matrix modification by adding nano-reinforcements, such as nanoparticles [13,14] and carbon nanotubes (CNTs) [15,16,17], is a viable way to improve composite performance. As an example, CNTs are capable of enhancing composite mechanical properties [18] and damping [15,16,19], but they are expensive and may be hard to handle.

Nanofibrous mat interleaving is a simple and smart approach: it can be virtually applied to any commercial prepreg during lamination, without the need for preliminary resin modification [20,21]. Nanofibrous nonwovens can be applied to composites for enhancing mechanical performance, mainly interlaminar fracture toughness [22,23,24,25,26,27], or adding new properties [28,29]. The use of such nano-reinforcements in composite materials is interesting because they are lightweight and can be integrated only where most required, according to an accurate lamination design, thus contributing to limit overall weight increase, as well as limiting the lowering of thermal and mechanical properties. While the use of thermoplastic nanofibers, as such as Nylon and poly(ε-caprolactone) (PCL), are well renowned to nano-modify laminates for hindering delamination [30], such nonwovens have a limited effect on damping enhancement [31,32].

Rubbery nanofibrous mats are new promising materials for increasing both interlaminar fracture toughness and damping of composite laminates, but there is no report about their actual use. At present, only a previous work by the authors on CFRP laminate modification by rubbery nanofibers can be found in the literature [31]. The lack of reports is surely connected to difficulties in obtaining rubber-made or rubber-containing nanofibers. Indeed, most of the reported works are just proofs-of-concept or deal with micrometric fibers [33,34,35,36,37], or they concern the mandatory the crosslinking of rubber during or after the electrospinning process [36,37,38,39,40]. At room temperature, liquid rubber, i.e., not crosslinked, is above glass transition: its cold flow [41], indeed, prevents keeping any defined structure, including nanofibers, leading to complete loss of nanostructured morphology. A work [42] presents the possibility of electrospinning nitrile rubber mixed with a thermoplastic polymer, aiming at modifying the toughness of pure resin. However, fundamental mechanical and thermomechanical characterizations of the nano-modified material is lacking.

Recently, the authors reported the production of dimensionally stable rubbery nanofibrous mats made of nitrile butadiene rubber and poly(ε-caprolactone) (NBR/PCL) blends [43]. The attained nanofibers can hold up to 60 wt% of uncrosslinked NBR: the resulting nanofibrous mats show elastomeric behaviour, still maintaining good handleability necessary to their placement between prepregs [43]. Our previous works were focused on the production and characterization of such rubbery nanofibers [43], as well as preliminarily exploring their integration into CFRP laminates for hindering delamination and potentially enhancing damping [31], reaching interesting positive results.

In the present study, a deeper investigation on the effect of such rubbery nanostructured materials on the damping of epoxy CFRP laminates is presented. In particular, the effect of the number and positioning of modified interleaves, as well as the amount of rubbery material (using membranes with different grammage), are investigated through dynamic mechanical analysis (DMA) by analysing tanδ behaviour. Moreover, double cantilever beam (DCB) specimens modified with mats having different thickness were produced and tested for evaluating how the interleaved mat grammage influences the CFRP interlaminar fracture toughness.

**Scheme 1:**
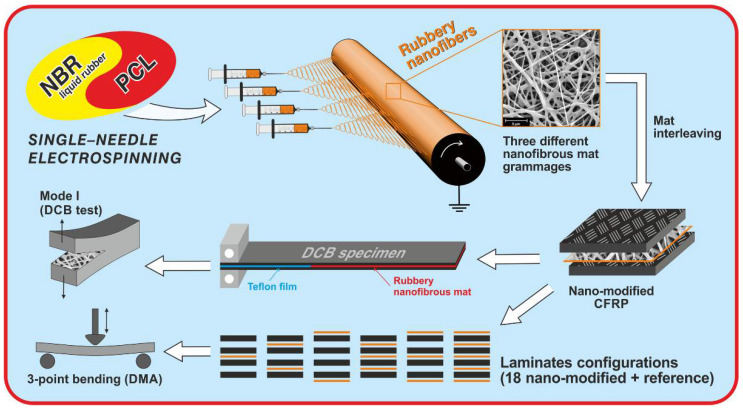
Sketch of the paper rationale: NBR/PCL blend nanofibers production by single-needle electrospinning process, mats integration in laminated CFRPs, and their testing for damping and Mode I interlaminar fracture toughness evaluation.

Scheme 1 depicts a sketch of the paper rationale.

## 2. Materials and Methods

### 2.1. Materials

Carboxylated nitrile butadiene rubber (NBR) Nipol 1072CGX was purchased from Zeon Chemicals (Dusseldorf, Germany) (68 mol% butadiene (Bu), 28 mol% acrylonitrile (ACN), 4 mol% methacrylic acid (MAA)). Poly(ε-caprolactone) (PCL), M_w_ 70,000–90,000, was purchased from Sigma-Aldrich (St. Louis, MO, USA). Polymers were both used without any preliminary treatment. Nylon 66 (Zytel E53 NC010) was kindly provided by DuPont (Wilmington, DE, USA). *N,N*-dimethylacetamide (DMAc), chloroform (CHCl_3_), formic acid and trifluoroacetic acid (TFA), were purchased from Sigma-Aldrich and were used without any preliminary treatment. Plain weave carbon fabric (200 g/m^2^) in epoxy matrix prepreg (GG204P IMP503Z-HT) for composite lamination was supplied by G. Angeloni s.r.l. (Venezia, Italy).

### 2.2. Solutions, Blend Preparation and Nanofibrous Mats Electrospinning

NBR/PCL 60/40 blend was prepared by mixing NBR and PCL solutions in the proportion of 60 wt% and 40 wt%, respectively. Polymer blends were stirred at room temperature for minimum 2 h to ensure proper homogenization. NBR solution, 10 wt%, was prepared in DMAc (e.g., 1.0 g of polymer in 9.6 mL of solvent) under magnetic stirring at room temperature until the formation of a homogeneous solution. PCL solution, 10 wt%, was prepared in CHCl_3_/DMF 1:1 wt (e.g., 1.0 g of polymer in 3.0 mL of CHCl_3_ and 4.8 mL of DMF) under magnetic stirring at room temperature until the formation of a homogeneous solution.

Rubbery nanofibrous mats containing 60 wt% of NBR (*n*-60/40) were produced using an electrospinning machine (Lab Unit, Spinbow s.r.l., Bologna, Italy) equipped with four 5 mL syringes joined to translating needles (length 55 mm, internal diameter 0.84 mm) via Teflon tubing. Electrospinning parameters were: flow rate 0.60 mL/h, electric potential 18.5 kV, distance 13 cm, electrostatic field 1.4 kV/cm, temperature 20–21 °C, RH 21–24%. Fibers were collected on a rotating drum (tangential speed of 0.39 m/s), covered with oven paper to favour supporting removal, until reaching 10, 20 and 40 μm of thickness. The mat thickness was measured with an analog indicator (Borletti, Italy), under 360 g/m^2^ pressure; at this measuring condition, the produced mat grammages were roughly equivalent to 5, 10 and 20 g/m^2^, respectively. The mats had final dimensions of 20 cm × 40 cm. For each mat thickness, the corresponding grammage was evaluated by averaging measurements done on three pieces of 5 cm × 5 cm. Table 1 reports the characteristics of the produced *n*-60/40 nanofibrous mats.

Nylon 66 and PCL nanofibrous mats were exclusively produced for comparing their mechanical properties with the rubbery ones. Nylon 66 was electrospun from a 13 wt% solution, prepared in a TFA/formic acid/chloroform 10:60:30 vol%. solvent system as reported in reference [44]. Electrospinning parameters were the same as reported for producing Ny250 mat in reference [44]. PCL membrane was produced according to the procedure reported in reference [43].

Nanofibrous mats were analyzed by scanning electron microscopy (Phenom ProX, ThermoFisher Scientific, Waltham, MA, USA) to determine nanofibers morphology. All analysed surfaces were gold coated to make them conductive. Average diameter values were calculated from at least 100 diameter measurements, manually done on single nanofibers by means of the Photoshop measurement tool.

### 2.3. Tensile Testing of Rubbery Nanofibrous Mats

Tensile tests of nanofibrous mats were made using a universal testing machine (Remet TC-10, Bologna, Italy) equipped with a 10 N load cell, and tested at 10 mm/min crosshead separation rate. Nanofibrous membranes were anchored in a paper frame, as previously reported [44], fixing them with cyanoacrylate glue (Super Attack, Loctite, Germany) for better handling and avoiding any mat slippage. Paper frames were 47 mm × 67 mm and 25 mm × 45 mm outer and inner dimensions, respectively. Before starting the test, the paper frame was cut. After the tensile test, the specimen was recovered and weighted for the stress (σ) calculation according to Equation (1).

Load data were normalized adopting a method based on the specimen mass normalization instead of its cross-section area put forward by the authors [44], to discard the influence of voids, thus leading to reliable stress–strain curves. Stress (σ) data were obtained according to the following equation:

(1) $$\sigma_{eq}=\rho_{m}\frac{F}{m}L$$

where $\rho_{m}$ is the material density (i.e., in the present case, the density of NBR/PCL 60:40 wt polymer blend, 1.145 g/cm^3^), *m* is the specimen mass, *L* is the specimen initial length, and *F* is the force. At least five specimens were tested for each nanofibrous mat grammage. Tensile test data were also analyzed by means of a fitting model [44,45]:

(2) $$\sigma \left(\varepsilon \right)=a\varepsilon +b-be^{-c\varepsilon }=a\varepsilon +b\left(1-e^{-c\varepsilon }\right)$$

where *a*, *b* and *c* are parameters experimentally determined to obtain the data fitting. Details on the superior reliability of the mass-based load normalization method are deeply investigated in reference [44].

### 2.4. CFRPs Production and Characterization

CFRP laminates were prepared via hand lay-up in an air-conditioned room (21–23 °C, 23–26% RH). A preliminary mild heat treatment (2 h at 45 °C under vacuum) was applied before the curing cycle for better impregnation of the nanofibers. The CFRP laminates were consolidated in an autoclave for 2 h at 135 °C, under vacuum, 6 bar external pressure, with a heating/cooling ramp of 2 °C/min.

The nanofibrous mats were added during the lamination step, by applying the mat (supported on the paper substrate) on the prepreg surface, and then removing the supporting paper before adding other prepreg plies.

Specimens for interlaminar fracture toughness evaluation via a Double Cantilever Beam (DCB) were prepared stacking 14 prepreg plies, interleaving a nanofibrous mat in the central interface, and using a Teflon film as crack trigger, according to ASTM D5528-01. An unmodified laminate was also produced as a reference. Details of DCB specimens are reported in Table 2.

DCB tests were carried out using a two-column hydraulic universal testing machine (Remet TC-10, Bologna, Italy) equipped with a 1 kN load cell and tested at 3.0 mm/min crosshead separation rate. The energy release rate for Mode I loading (G_I_, in J/m^2^), both at the initial and propagation stages (G_I,*C*_ and G_I,*R*_, respectively), was evaluated using Equation (3), according to ASTM D5528-01 [46]:

(3) $$G_{I}=\frac{3P\delta }{2ba}1000$$

where *P* is the load (in N), *δ* the crosshead displacement (in mm), *a* the crack length (in mm), and *b* is the specimen width (in mm). The G*_R_* was evaluated considering a crack length range of 47–90 mm. After the DCB test, specimens micrographs were recorded by optical microscope (Zeiss Axioscope, Jena, Germany) for analysing crack path, as well as delamination surfaces for investigating matrix behaviour via SEM microscopy (Phenom ProX, ThermoFisher Scientific, MA, USA).

**Figure 1:**
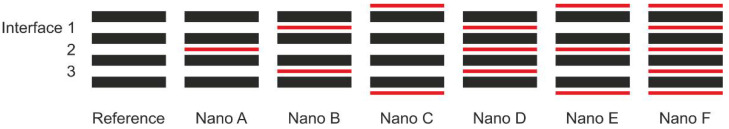
Schematic representation of the different laminate configurations for DMA tests (black lines: CFRP ply, red lines: nanofibrous mat). The nano-modified configurations are replicated for the three different membrane grammages.

Specimens for DMA tests were prepared stacking 4 plies of prepregs. Final specimens have dimensions of 30 mm × 8 mm. The interfaces were nano-modified according to the scheme reported in Figure 1. An unmodified reference specimen was also produced for the sake of comparison.

While it is impossible at the present time to provide exact information about the real content of rubbery materials into the nano-modified laminates, it can be safely assumed that, in the worst case, the loading percentage should not exceed the 5–10 wt% with respect to the resin fraction.

Dynamic mechanical analysis (DMA) was carried out with a DMA 242 E Artemis (Netzsch, Selb, Germany) instrument in three-point bending deformation mode, using a 20 mm fixed span. DMA analyses in a temperature ramp were carried out in the −50 + 230 °C range at a 3 °C/min heating rate, 1 Hz frequency, amplitude 20 μm, and static force/dynamic force ratio = 1.5.

## 3. Results and Discussion

Fracture toughening improvement of laminated CFRPs by rubber addition is a well-established approach. On the contrary, the interleaving of rubbery nanofibrous mats is a new way to add the same benefits of rubber toughening, but localized only on the most required critical component regions, possibly contributing to limiting detrimental rubber effects on the other composite mechanical properties (i.e., elastic modulus and strength).

Our previous work [31] demonstrated the dramatic effect of NBR/PCL rubbery nanofibrous mats on the interlaminar fracture toughness and the overall CFRP thermomechanical properties. The boost in the energy release rate (G) was outstanding: in the most favourable case, the energy required for the crack to initiate and propagate was +480% and +340%, respectively. It was established that both the number and type of interleaved mats, i.e., the percentage of rubber in the blend nanofiber, affect the laminate thermomechanical behaviour. Preliminary data suggested that a high number of nano-modifications and a high amount of NBR lead to a significant improvement of the composite damping, thanks to NBR/PCL mixing with the epoxy resin. Indeed, NBR and PCL may diffuse into the epoxy resin during the curing cycle, leading to a toughened matrix. Consequently, the energy dissipation window of the modified composites widens down toward lower temperatures, thanks to the presence of the highly damping NBR component. However, the topic needs further investigations to better show the evidence of the effect of the number and position of interleaved nanofibers, as well as the mat grammage.

In the present study, rubbery mats with 60 wt% of NBR (*n*-60/40) were chosen to maximize the toughening effect. To thoroughly assess the damping behaviour as a function of number, position and amount (grammage) of nano-modifications, different laminate configurations were studied. Indeed, the effect is expected to be in some way proportional to the modification extent (number and grammage of mats), besides dependent on the position of the inserted nano-modification(s). For these reasons, a 4-ply CFRP was selected for limiting the possible combinations of all these parameters, thus allowing testing a wide spread of different cases (18 configurations + reference CFRP, see Figure 1).

### 3.1. Nanofibrous Mat Characterization: Morphology, Grammage and Tensile Properties

**Figure 2:**
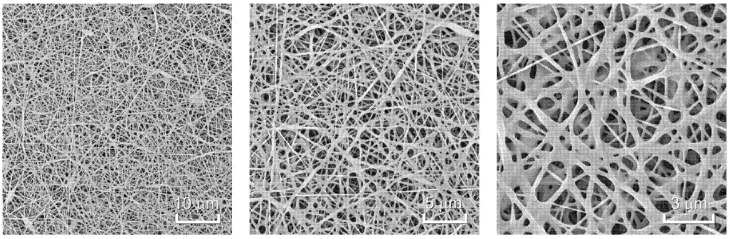
SEM micrographs of electrospun *n*-60/40 nanofibers (only *n*-60/40_20 mat is shown for brevity).

Electrospun rubbery mats show a nanofibrous morphology with only very limited filming phenomena (Figure 2), thanks to the ability of NBR/PCL miscible blend to behave as thermoplastic elastomers (TPEs), as previously demonstrated [43]. It was also found that such morphology is kept at least for 2 years after production at common ambient conditions. Nanofibers have diameters in the 200–300 nm range (Table 1), and no differences are found on the nanofibrous structure by varying the final mat grammage, as expected.

Nanofibers are arranged in a random disposition to facilitate their impregnation with the resin and impart a reinforcement as much as possible isotropic on the plane. Actually, to avoid the latter issue, the random disposition is strictly required for nanofibers that do not melt or “fluidize” during the curing cycle, like high-T_m_ Nylon [45] or high-T_g_ polyaramid [47]. In the present case, the NBR/PCL blend fluidizes upon curing, allowing its mixing with the epoxy, leading to the formation of an almost homogeneous toughened matrix localized region, as previously demonstrated [31]. However, the random disposition is preferable for obtaining an optimal integration of the nonwoven and to avoid the insertion of a too compact membrane.

**Figure 3:**
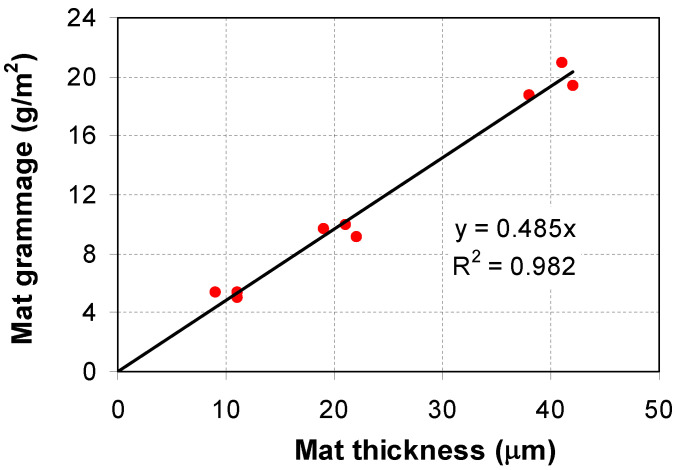
Grammage–thickness relationship of the produced *n*-60/40 mats.

For each laminate configuration, three different nanofibrous mats characterized by different thickness were interleaved for studying their effect on laminate properties. Since the thickness measurement of such soft materials may be inaccurate and, most importantly, dependent on the pressure measurement, the mat grammage will be taken into consideration instead. The interleaved mats have grammages of about 5, 10 and 20 g/m^2^, whose relationship with measured mat thickness is reported in Figure 3. The highlighted linear relation holds, however, solely in the case that the same measurement system is adopted, i.e., the nanofibrous mat sample is subjected to the same pressure during thickness evaluation [44].

**Figure 4:**
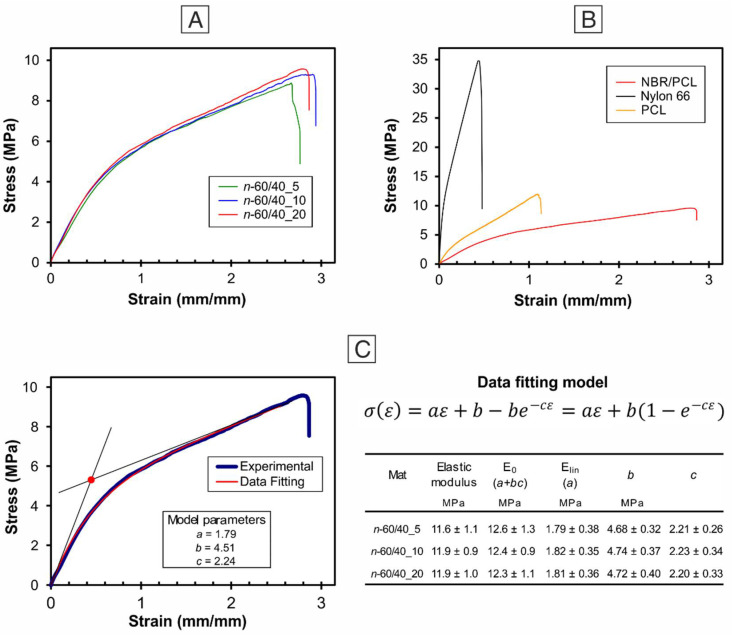
(**A**) Stress-strain curves of *n*-60/40 nanofibrous mats with different grammage; (**B**) comparison of tensile behaviour of rubbery mat with common thermoplastic ones; (**C**) example of data fitting application and relevant data deriving from stress–strain curve analysis. Stress data were calculated using a reliable mass-based load normalization, according to Equation (1).

Tensile tests were carried out on the three different *n*-60/40 mats to verify mat performance consistency, which is a prerequisite for further comparing the CFRP results. Thanks to the superior reliability of the adopted method for normalizing load data, the obtained stress–strain curves are almost superimposed (Figure 4A), as also demonstrated in our previous specific work [44]. Indeed, this normalization is based on the specimen mass (Equation (1)) instead of its cross-section area, thus allowing to overcome any issue deriving from inaccuracies of mat thickness evaluation. Since mat voids are discarded, the stress (σ), as defined in Equation (1), describes the stress equivalent to a specimen with the same gauge length, width and mass, but condensed in a bulk film.

*n*-60/40 mats show ductile behaviour, due to the fibrous nature, with outstanding deformation at the break, which reaches 280–300% (Figure 4A). The comparison of its behaviour with the ones displayed by “standard” and well-known nanofibrous mats, such as Nylon 66 and PCL, is shown in Figure 4B. The outstanding rubbery mat deformation, several times the ones that characterize polyamide and polyester membranes, is clearly evident: it is surely ascribable to the NBR liquid rubber, which confers an elastomeric behaviour to the whole nanofibrous structure. Conversely, stiffness and strength are reduced, especially if compared with the Nylon 66 mat. Stress–strain curves were also analyzed using a data fitting model previously introduced by the authors [44], allowing a deeper insight into tensile testing data. Figure 4C displays an example of data fitting. The analysis of the fitting parameters *a*, *b* and *c* allows us to define two elastic moduli: E_0_, resembling the “classic” elastic modulus, is associated with the mat stiffness at early strains, while E_lin_ accounts for stiffness at high strains. A complete explanation of the fitting model can be found in references [44,45].

### 3.2. Damping Evaluation of CFRP Laminates via Analysis of Tanδ Behaviour

Nanofibrous mats having such elastomeric characteristics may strongly impact the thermomechanical behaviour of modified CFRP laminates. Dynamic mechanical analysis (DMA) can provide information about the dynamic behaviour of materials, such as damping ability, by evaluating tanδ behaviour. Indeed, the tanδ value, or the “damping factor”, represents the ratio between E’’ and E’ (loss and storage moduli, respectively), accounting for the material ability to dissipate energy. Moreover, this test allows for evaluating the temperature range in which the damping is prevalent.

**Figure 5:**
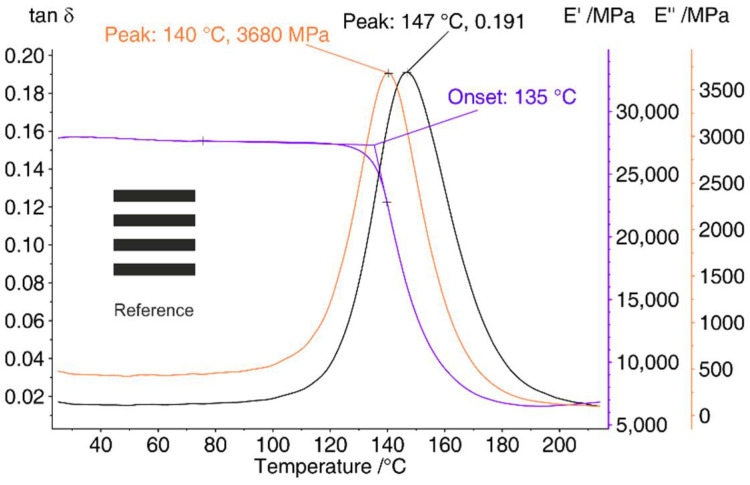
DMA of reference laminate (C-Ref): E′ (purple), E″ (orange), tanδ (black).

Figure 5 reports the DMA of the reference CFRP (C-Ref) without interleaved mats, characterized by an onset of E’ drop of 135 °C and a tanδ peak at 147 °C. As expected for stiff materials, below glass transition the damping is low (tanδ = 0.015 ± 0.002).

For the nano-modified samples, only the tanδ profile will be reported, as the tanδ behaviour is the focus of the present investigation.

**Figure 6:**
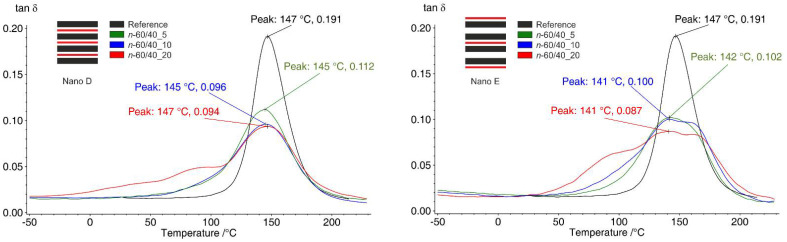
Comparison of tanδ curves of Nano E (**left**) and Nano D (**right**) laminate configurations.

Nano D and Nano E configurations have the same number (3) of interleaved mats, but located in different positions (Figure 6).

Nano D has all the internal interfaces modified, while in Nano E the two outers plus the central one are modified. Tanδ peak broadening is associated with a widening of the relaxation temperature range in which the material can dissipate energy (damping). This phenomenon is evident for the presented samples, especially for the highest grammage modification. It is likewise evident that the different lamination sequences display a different tanδ peak broadening, highlighting the importance of the lamination design.

The damping of rubber-containing mats is higher than C-Ref also at low temperature, suggesting a general improvement of the composite vibration dampening, especially for the Nano D configuration, with the highest mat grammage. A stronger dependence of tanδ values on interleaved mat grammage in Nano E can be observed, while the highest grammage adds a significant boost of damping also at low temperatures in the Nano D configuration.

The damping of rubber-containing mats is higher than C-Ref also at low temperature, suggesting a general improvement of the composite vibration dampening, especially for the Nano D configuration, with the highest mat grammage. It can be observed that there is a stronger dependence of tanδ values on the interleaved mat grammage in Nano E, while the highest grammage adds a significant boost of damping also at low temperatures in the Nano D configuration.

**Figure 7:**
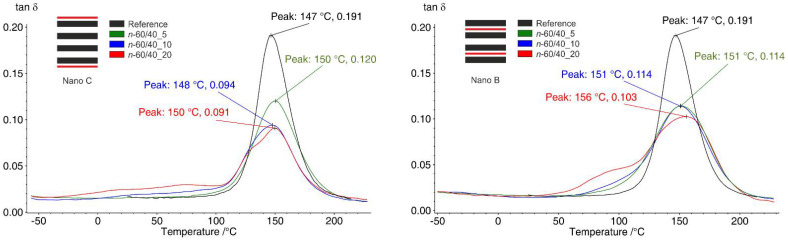
Comparison of tanδ curves of Nano C (**left**) and Nano B (**right**) laminate configurations.

Nano C has a smaller number of nano-modifications than the two previously discussed Nano D and Nano E configurations; therefore, it shows lower damping improvement at lower T, which is almost independent of the mat grammage regarding peak broadening (Figure 7). By contrast, a positive effect at low temperature is still present for CFRPs with 10 and 20 g/m^2^ mats.

By comparing Nano C with Nano B, which has the same number of interleaved mats, some differences are found (Figure 7). The latter configuration shows a higher broadening of the tanδ peaks: the modification of inner interfaces seems to be more effective than outer ones. This case also suggests that modification of the outer laminate layers determines a lower impact on the CFRP damping with respect to inner nano-modifications.

**Figure 8:**
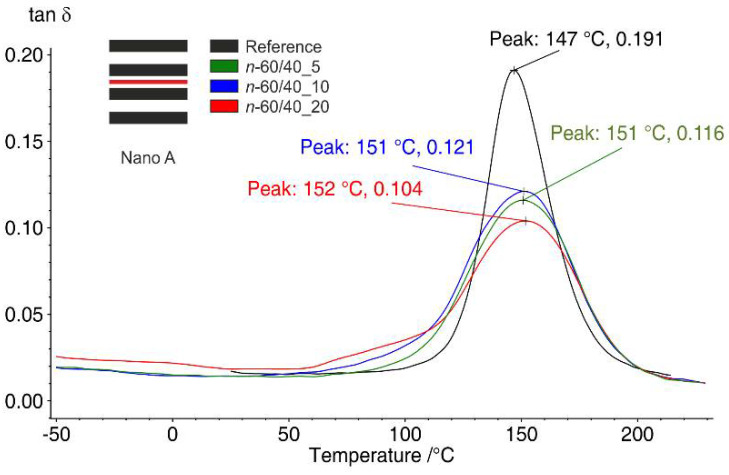
Tanδ curves of Nano A laminate configuration.

When it comes to a single nano-modified layer (Nano A), while still showing a slightly positive effect on tanδ, it is clear that the overall damping improvement is limited if compared to previous cases (Figure 8). However, it is noticeable that the performance of Nano A laminate is quite similar to Nano C, even though the latter presents two interleaved mats. Again, it confirms that the modification of the outer plies seems to be less effective.

**Figure 9:**
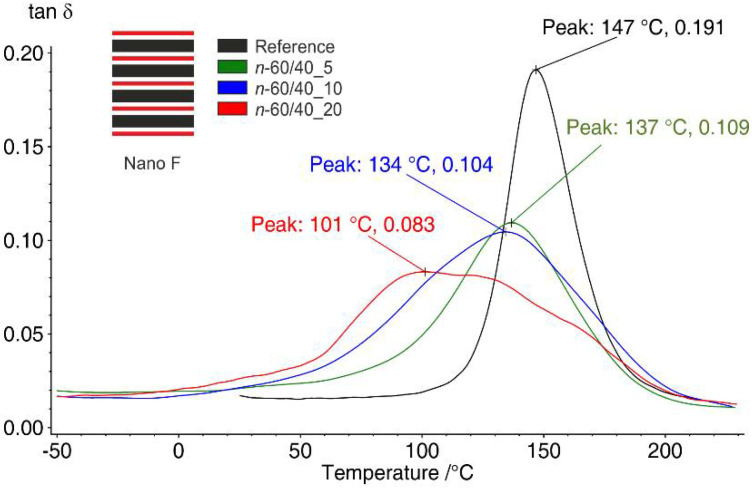
Tanδ curves of Nano F laminate configuration.

The fully nano-modified Nano F configuration shows, obviously, the highest broadening of the tanδ curve, together with the highest enhancement of tanδ also at low temperatures (Figure 9).

Moreover, the overall effect of such nano-modifications appears to be more extended than the improvement carried out by both Nano B and Nano E laminate configurations virtually combined together.

**Figure 10:**
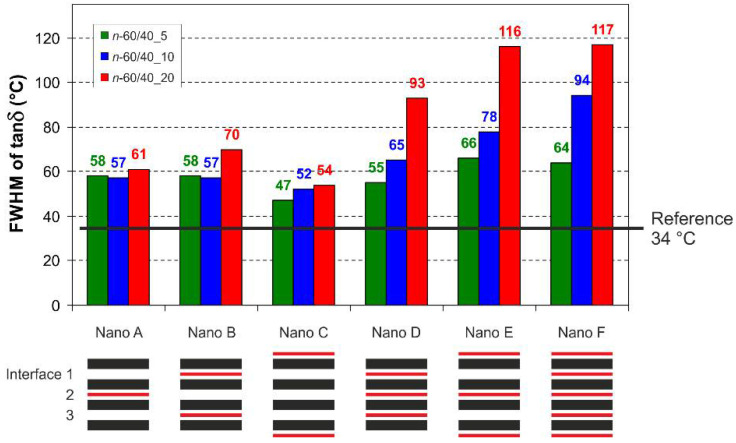
Comparison of Full Width at Half Maximum (FWHM) of tanδ peaks (standard deviations, not reported, are not higher than 3 °C in all the cases).

To better define the tanδ peak broadening, in Figure 10 the measured Full Width at Half Maximum (FWHM) of the tanδ peaks for each tested laminate configuration are reported. All the nano-modified laminates show some broadening of the tanδ peak. Even in the worst-case scenario (Nano C, *n*-60/40_5), the increase in FWHM is significant (47 °C vs. 34 °C of the reference C-Ref). In general, an increment of the FWHM occurs by increasing both the number of nano-modifications and the mat grammage. By interleaving two mats (Nano B and Nano C), the results in terms of FWHM is quite similar to the use of a single mat (Nano A), almost independently of the mat grammage. Instead, regarding CFRPs with three nano-modifications (Nano D and Nano E), as well as the full modified one (Nano F), the effect of mat thickness is amplified, resulting in higher FWHMs for increased mat grammage.

**Figure 11:**
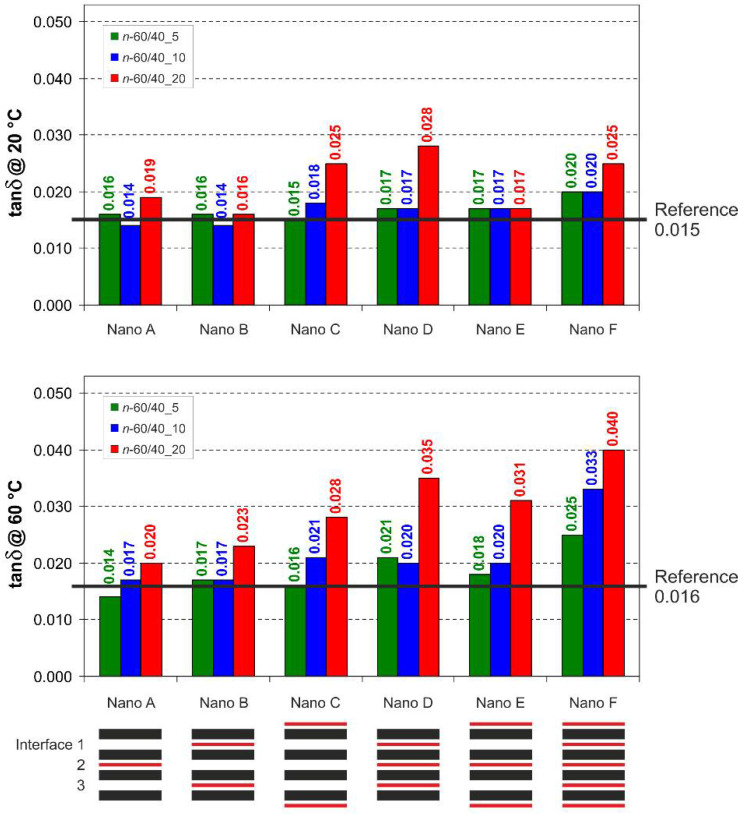
Tanδ and values at 20 and 60 °C (standard deviations, not reported, are not higher than 0.002 in all the cases).

Tanδ values reported in histograms of Figure 11 allow to better compare the CFRP damping at specific temperatures, like at ambient temperature (20 °C) and near the PCL melting temperature (60 °C [43]). At 20 °C, only the configurations with the highest mat grammage show some improvement of the tanδ, except for the Nano E sample, while at 60 °C the enhancement is generally more pronounced.

**Figure 12:**
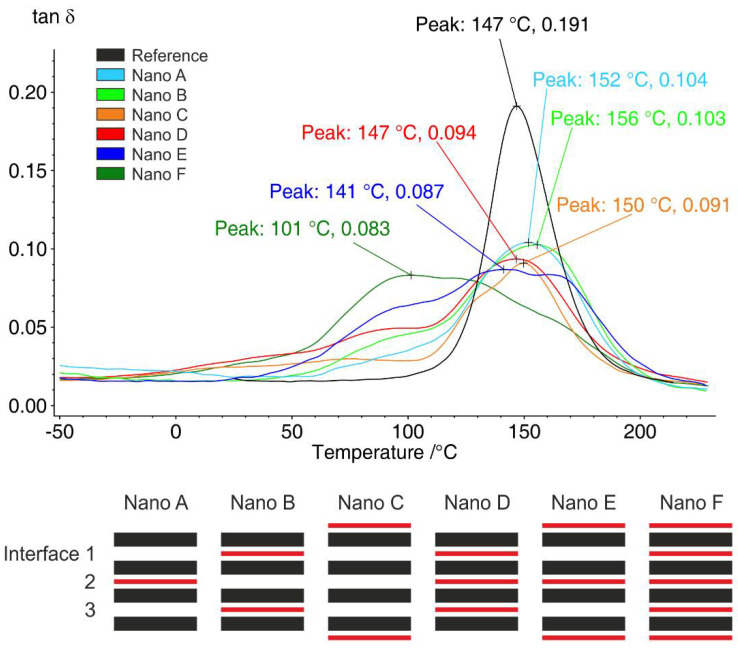
Comparison of tanδ curves for all tested laminate configurations nano-modified with *n*-60/40_20 mats.

Figure 12 shows a comparison of all nano-modified laminate configurations with *n*-60/40_20 mats, as well as the unmodified CFRP, to quickly compare their tanδ trends.

DMA shows a strong effect on tanδ behaviour when the highest mat thickness (40 μm, *n*-60/40_20) is interleaved. By contrast, a 10 μm mat thickness (*n*-60/40_5) shows a slight impact on overall damping improvement, even when all the interfaces are nano-modified. Intermediate thickness (20 μm, *n*-60/40_10) affects tanδ behaviour in between the two extremes.

### 3.3. Mode I Interlaminar Fracture Toughness: DCB Tests, Crack Path and Delamination Surfaces Characterization

Rubbery nanofibrous mats, as previously demonstrated [31], contribute significantly to enhance interlaminar fracture toughness: mats with 40 μm thickness (17–20 g/m^2^) boost G_I_ up to +480%.

While our previous study focused on the impact of rubber fraction in the NBR/PCL blend, the present work investigates the effect of *n*-60/40 mat grammage on laminate damping. An enhanced CFRP damping leads to a less fragile behaviour of the epoxy matrix, since it may locally deform viscoelastically more easily. Consequently, a matrix with improved local ductility may be able to hinder crack propagation and delamination, in particular when only applied at critical regions, where cracks are more prone to originate.

**Figure 13:**
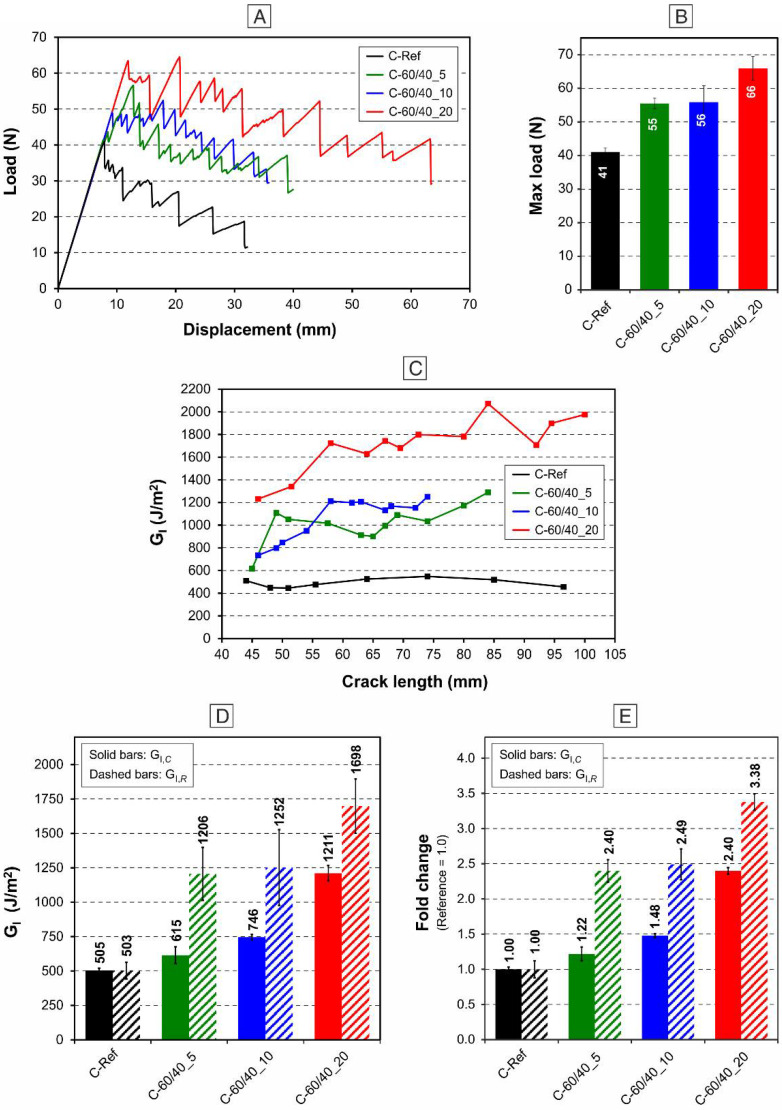
DCB tests results: (**A**) load–displacement curves; (**B**) maximum load; (**C**) *R*-curves related to the same specimens displayed in (**A**); (**D**) average G_I,*C*_ and G_I,*R*_; (**E**) G_I_ fold change (bars are expressed as the relative variation of the value with respect to the reference sample (C-Ref), whose value is set as 1.0).

DCB test results are summarized in Figure 13. The superior ability of rubbery-modified laminates at contrasting delamination is evident. Load-displacement curves (Figure 13A), even though they did not consider crack advancements, give a rough indication of the hinderance effect in crack propagation ability: modified laminates show trends higher than reference, regardless of the actual mat grammage (35–60% of the improved maximum load, Figure 13B).

Analysis of *R*-curves (Figure 13C), i.e., G_I_ vs. crack length, confirms the trends displayed by load–displacement ones, showing the maximum effect on delamination hampering when the *n*-60/40_20 mat is interleaved (C-60/40_20). Histograms of G_I_ results help to better compare the performance achieved. While all the rubbery-modified laminates have a strong impact on G_I,*R*_, from +140% to +238%, the effect on G_I,*C*_ is more limited and more dependent on the interleaved mat grammage. The enhancement is +22%, +48% and +140% for laminates nano-modified with *n*-60/40_5, *n*-60/40_10, and *n*-60/40_20 mats, respectively. The results demonstrate that the highest mat grammage imparts the maximum enhancement of both G_I,*R*_ and G_I,*C*_ (C-60/40_20 sample). The laminate interleaved with medium-grammage mat (C-60/40_10) still shows good delamination performance, while the thinnest mat mainly affects G_I,*R*_ (C-60/40_5). By comparing the performance of C-60/40_20 and the one reported in our previous work [31], where the same mat grammage was used, it can be noticed that the found G_I_ enhancement is different: +140% vs. +480% in G_I,*C*_ and +238% vs. +340% in G_I_,*_R_*. It is to underline that the present laminates were produced using a prepreg with the same carbon fabric but characterized by different matrix systems (IMP503Z-HT vs. IMP503Z), which surely affects the performance achieved. For a discussion of the thermomechanical behaviour of the two different prepregs refer to reference [31].

**Figure 14:**
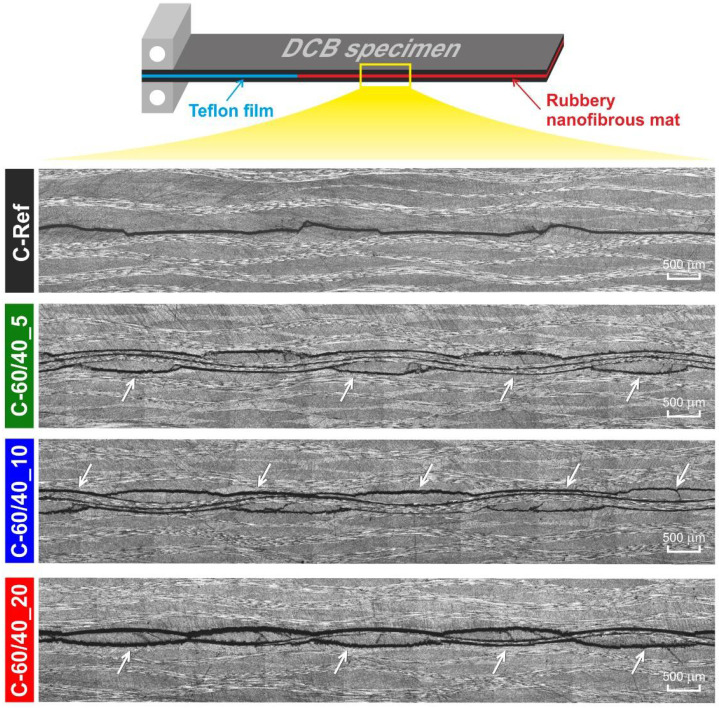
Micrographs of DCB specimens after the delamination tests. The arrows indicate the central plane where the Teflon film trigger is inserted.

Figure 14 shows micrographs of DCB specimens after tests for analysing crack path.

At first glance, rubbery-modified CFRPs display a significantly different behaviour with respect to the unmodified ones. Indeed, C-Ref is characterized by a crack propagation affecting only the designated interface, i.e., the central one where the Teflon film was inserted as crack trigger. In contrast, nano-modified laminates behave differently: crack paths appear more complicated and uneven. Even though some differences in Mode I performance are present, as revealed by the DCB results, fracture behaviour is quite similar to each other. All the rubbery-reinforced laminates display cracks propagated not only along the nano-modified interface, but also in adjacent ones, regardless of the mat grammage. This significant change in behaviour is indicative of strong interface reinforcement, which is responsible for the development of crack propagation along planes other than the central one. Indeed, the matrix toughening achieved is so effective that the crack, following the path of least resistance, propagates across the very stiff woven carbon fabric, breaking it.

**Figure 15:**
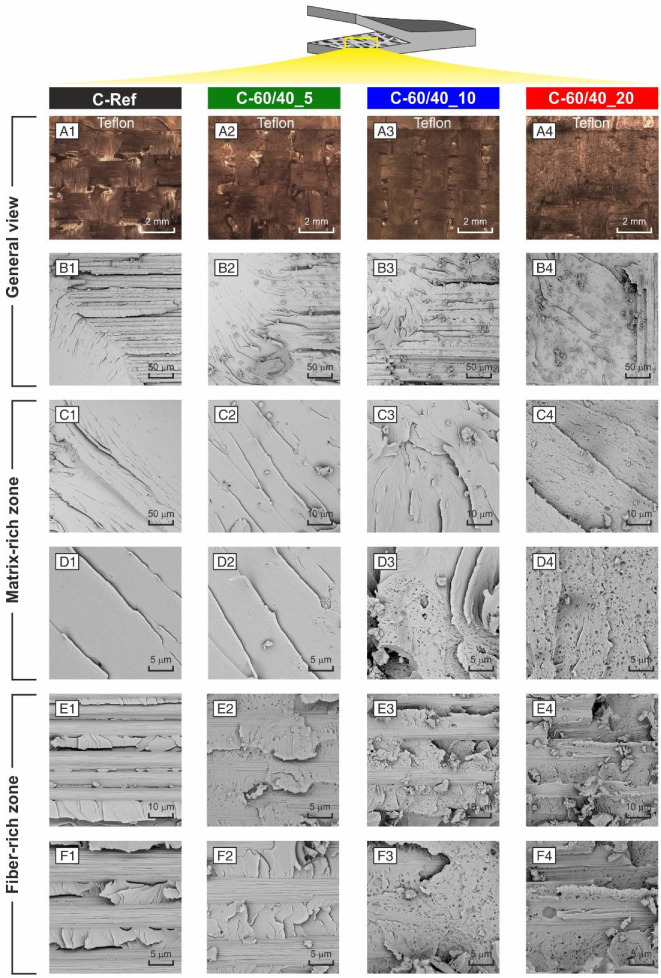
Optical and SEM micrographs of delamination planes of DCB specimens after testing: (**A1**–**A4**) and (**B1**–**B4**) images display the whole composite surface morphology, (**C1**–**C4**) and (**D1**–**D4**) images focuses on the epoxy matrix, (**E1**–**E4**) and (**F1**–**F4**) images highlight the interaction between fiber and matrix.

With the aim of better investigation of the the laminates behaviour, the delamination surfaces, after DCB tests, were also recorded (Figure 15).

As revealed by low magnification optical images (Figure 15A1–A4), the morphology of rubbery-modified CFRP surfaces appear different, suggesting an increasing toughening extent trend by increasing interleaved mat grammage. Indeed, while the carbon fabric pattern in C-Ref is clearly visible, it is scarcely detectable in C-60/40_20. The other nano-modified CFRPs present a behaviour between the two extremes. A deeper investigation was carried out via electron microscopy. Unmodified laminate (C-Ref) displays a brittle fracture behaviour typical of thermosetting epoxy resins: interlaminar resin pockets have fractures characterized by wide flat planes (Figure 15B1,D1). The same behaviour is visible close to carbon fibers, which appear almost resin-free (Figure 15E1,F1).

Analysing the fracture surfaces of the laminate with the highest rubbery blend fraction (C-60/40_20), the matrix morphology is completely different (Figure 15B4,D4): it displays a very rough surface, characterized by holes, due to plastic deformation (Figure 15D4). While in C-Ref carbon fibers are “clean” (Figure 15E1,F1), here they are almost completely embedded into the matrix (Figure 15E4,F4). It is worth mentioning that rubbery nanofibers are not visible because they completely lost the nanofibrous morphology during the curing cycle, carried out above the PCL melting temperature. The modification with NBR/PCL rubbery nanofibrous mats up to 60 wt% of rubber toughen the epoxy matrix without observing evident phase separation, which, instead, extensively happens when a bulk film or NBR is interleaved (see DCB delamination surfaces in ref. [31]).

The use of a half-grammage rubbery mat (C-60/40_10) leads to a less extended epoxy toughening, as displayed by Figure 15D3: the very rough matrix surfaces visible in C-60/40_20 sample is here less detectable. However, carbon fibers still result well embedded into the matrix. When the lowest mat grammage is used (C-60/40_5), the matrix nano-modification is limited (Figure 15C2,D2). Analysing matrix fracture, it resembles the one displayed by unmodified laminate. For what concerns carbon fibers appearance, they are similar to the ones present in C-Ref (Figure 15F2) and the ones visible in both C-60/40_10 and C-60/40_20 (Figure 15E2). Probably, the presence of not completely embedded carbon fibers accounts for the lower improvement of interlaminar fracture toughness revealed by DCB tests, even though still signifantly present in propagation (+140% of G_I,*R*_). SEM observations confirm the ability of NBR/PCL blend nanofibrous mats to toughen the epoxy resin, leading not only to improved laminate damping, but also to increased interlaminar fracture toughness.

## 4. Conclusions

CFRP laminates nano-modified via rubbery nanofibers interleaving is a recently introduced way to increase material damping and improved delamination resistance in well localized composite regions of a composite.

NBR/PCL rubbery nanofibrous mats with 60 wt% NBR were produced via single-needle electrospinning and interleaved into epoxy CPRP laminates. A 4-ply laminate configuration was chosen to test a wide number of possible nano-modification arrangements. To better describe the mat effect, three different mat grammages (5, 10 and 20 g/m^2^) were used.

The present investigation demonstrates that both mat grammage and positioning affect CFRP tanδ behaviour recorded by DMA tests. Rubbery-modified laminates show tanδ peak broadening associated with a widening of the relaxation temperature range in which the material can dissipate energy, thus leading to enhanced damping. The effect is higher when high grammage mats are integrated and the nano-modified interleaves are in the inner layers. In addition, the damping enhancement raises when the number of interleaved interfaces is higher.

DCB tests demonstrate that nano-modified laminates increased interlaminar fracture toughness. While an outstanding improvement of G_I,*R*_ can be found for all the tested reinforced laminates regardless the mat grammage (from +140% to +238%), the effect on G_I,*C*_ is more dependent on it. The laminate with the highest mat grammage exhibits the best performance (+140% of G_I,*C*_), even though the intermediate mat grammage adds a significant boost (+48%).

The obtained results disclose the great capability of NBR/PCL rubbery nanofibrous mats to improve CFRP damping and interlaminar fracture toughness. Moreover, CFRP damping can be tailored by choosing the number and positioning of nano-modified interleaves, as well as mat grammage. Indeed, this approach provides an easy tool to impart a specific modification of the critical laminate regions to prevent crack propagation, ensuring an intrinsic safety of the material, with a limited impact on weight, dimension and costs.

## Figures and Tables

**Table 1:** Characteristics of electrospun rubbery mats.

Nanofibrous Mat	NBR Content (wt%)	Mat Thickness (μm)	Mat Grammage (g/m^2^)	Nanofiber Diameter (nm)
*n*-60/40_5	60	10 ± 1	5.3 ± 0.3	258 ± 29
*n*-60/40_10	60	20 ± 2	9.6 ± 0.5	252 ± 38
*n*-60/40_20	60	40 ± 2	19.7 ± 1.1	265 ± 41

**Table 2:** Details of produced DCB specimens.

DCB Specimen	Nanofibrous Mat	Specimen Width (*b*) (mm)	Specimen Length (mm)	Initial Delamination Length (*a*_0_) (mm)
C-Ref C-60/40_5	– *n*-60/40_5	20	140	45
C-60/40_10 C-60/40_20	*n*-60/40_10 *n*-60/40_20			

## Data Availability

The data presented in this study are available on request from the corresponding author.
